# Vitamin D-associated genetic variants in the Brazilian population: Investigating potential instruments for Mendelian randomization

**DOI:** 10.7705/biomedica.6972

**Published:** 2024-03-31

**Authors:** Caroline de Souza Silverio, Carolina Bonilla

**Affiliations:** 1 Departamento de Medicina Preventiva, Faculdade de Medicina, Universidade de São Paulo, São Paulo, Brasil Universidade de São Paulo Departamento de Medicina Preventiva Faculdade de Medicina Universidade de São Paulo São Paulo Brazil; 2 Escola de Artes, Ciências e Humanidades, Universidade de São Paulo, São Paulo, Brasil Universidade de São Paulo Escola de Artes, Ciências e Humanidades Universidade de São Paulo São Paulo Brazil

**Keywords:** Vitamin D, genome-wide association study, polymorphisms, single nucleotide, vitamin D-binding protein, vitamin D_3_ 24-hydroxylase, 25-hydroxyvitamin D_3_ 1-alpha-hydroxylase, Brazil, vitamina D, estudios de asociación del genoma completo, polimorfismos de nucleótido simple, proteína de unión a la vitamina D, vitamina D_3_ 24-hidroxilasa, 25-hidroxivitamina D_3_ 1-alfa-hidroxilasa, Brasil

## Abstract

**Introduction.:**

Vitamin D is required for bone and mineral metabolism and participates in the regulation of the immune response. It is also linked to several chronic diseases and conditions, usually in populations of European descent. Brazil presents a high prevalence of vitamin D deficiency and insufficiency despite the widespread availability of sunlight in the country. Thus, it is important to investigate the role of vitamin D as a risk factor for disease and to establish causal relationships between vitamin D levels and health-related outcomes in the Brazilian population.

**Objective.:**

To examine genetic variants identified as determinants of serum vitamin D in genome-wide association studies of European populations and check whether the same associations are present in Brazil. If so, these single nucleotide polymorphisms (SNPs) could be developed locally as proxies to use in genetically informed causal inference methods, such as Mendelian randomization.

**Materials and methods.:**

We extracted SNPs associated with vitamin D from the genome-wide association studies catalog. We did a literature search to select papers ascertaining these variants and vitamin D concentrations in Brazil.

**Results.:**

*GC* was the gene with the strongest association with vitamin D levels, in agreement with existing findings in European populations. However, *VDR* was the most investigated gene, regardless of its non-existing association with vitamin D in the genomewide association studies.

**Conclusions.:**

More research is needed to validate sound proxies for vitamin D levels in Brazil, for example, prioritizing *GC* rather than VDR.

## Resemen

**Introducción.:**

La vitamina D es necesaria para el metabolismo óseo y mineral, y participa en la regulación de la respuesta inmunitaria. También está relacionada con enfermedades crónicas en poblaciones europeas. En Brasil, existe una prevalencia elevada de deficiencia e insuficiencia de vitamina D, a pesar de la amplia disponibilidad de luz solar. Por lo tanto, es importante investigar el papel de la vitamina D como factor de riesgo de diversas enfermedades y establecer relaciones causales entre los niveles de vitamina D y los problemas de salud en la población brasileña.

**Objetivo.:**

Examinar variantes genéticas relacionadas con la vitamina D sérica en estudios de asociación genómica de poblaciones europeas y comprobar si estas mismas están presentes en Brasil. De ser así, estos SNPs podrían utilizarse como *proxies* en métodos de inferencia causal, tales como la aleatorización mendeliana.

**Materiales y métodos.:**

A partir del catálogo de estudios de asociación de genoma completo se extrajeron SNPs relacionados con los niveles de vitamina D. Luego se hizo una búsqueda bibliográfica para identificar los artículos que evaluaran estos SNPs y la concentración de vitamina D en Brasil.

**Resultados.:**

*GC* fue el gen más fuertemente asociado con los niveles de vitamina D, en concordancia con los resultados existentes en poblaciones europeas. Sin embargo, el gen *VDR* fue el más investigado, aunque no esté vinculado con la vitamina D en los estudios de asociación de genoma completo.

**Conclusiones.:**

Se necesita más investigación para validar *proxies* genéticos de los niveles de vitamina D en Brasil y se recomienda priorizar el gen *GC* en lugar de VDR.

Vitamin D is a steroid hormone and a fat-soluble vitamin required by the human body for physiological bone and mineral metabolism ^1^. It plays a role in immune response regulation ^2^, among other functions. When vitamin D levels are low, its insufficiency or deficiency may contribute to various adverse health outcomes, from skeletal disorders such as rickets and osteomalacia to extraskeletal conditions like cancer, infections, and cardiovascular, autoimmune, and neuropsychiatric diseases ^3^. However, evidence of a causal effect is still scarce for many of these health problems.

The main source of vitamin D is sunlight. Pre-vitamin D_3_ is converted from 7-dehydrocholesterol by ultraviolet radiation (UVR) B in the skin and then transported to the liver and other tissues to be metabolized to 25-hydroxy-vitamin D (25OHD) -the major circulating form- by the enzyme CYP2R1. The 25OHD is then further metabolized to 1,25 dihydroxy-vitamin D (1,25(OH)_2_D), primarily in the kidney, by the enzyme CYP27B1. The 1,25(OH)_2_D is the active metabolite of vitamin D, responsible for most of its biological actions achieved via binding to a specific nuclear vitamin D receptor (VDR) and eliciting the transcriptional regulation of target genes. The inactivation and catabolism of 25OHD and 1,25(OH)_2_D are carried out by the enzyme CYP24A1. Circulation in the bloodstream of pre-vitamin D_3_ and vitamin D metabolites occurs using the vitamin D binding protein (VDBP) and albumin ^4^.

The US Endocrine Society has defined concentrations of 25OHD above 30 ng/ml as sufficient, between 20 and 30 ng/ml as insufficient, and below 20 ng/ml as deficient vitamin D levels, or their equivalent in nmol/L (1 ng/ml=2,5 nmol/L). Cut-off values may differ between studies depending on whether they follow the recommendations of the US Endocrine Society, the US Institute of Medicine (12 ng/ml and 20 ng/ml as the thresholds for deficiency and sufficiency, respectively), or the UK Scientific Advisory Committee (below 10 ng/ml is considered vitamin D deficiency) ^1^^,^^5^. The proposed minimum thresholds are defined by criteria including the suppression of parathyroid hormone secretion, increased calcium absorption, good musculoskeletal health, and reduced fractures and falls ^1^.

Identifying causal associations of vitamin D with disease using observational methods can be difficult because of confounding variables and other biases often afflicting these studies. Some reports devise Mendelian randomization as a method to improve causal inference in epidemiology by employing genetic variants strongly associated with an exposure, known in this context as instrumental variables, which are unlikely to suffer the same observational biases ^6^. Mendelian randomization has become quite popular in the last decade, clarifying cause-and-effect relationships between many risk factors and disease outcomes ^7^. However, this success has been limited to populations of European descent, where most research is conducted. For Mendelian randomization to be effectively applied in Brazil (and other non-European populations) we need to select genetic variants that are instrumental variables for exposures in the local populations.

For that reason, we investigated single nucleotide polymorphisms (SNPs) strongly associated with serum vitamin D, initially detected in Europeans, to assess whether they could be used as proxies for vitamin D in the Brazilian population to determine causal relationships between vitamin D levels and chronic diseases using Mendelian randomization.

## Materials and methods

Single nucleotide polymorphisms associated with 25OHD (from now on, vitamin D) levels in blood were identified using the publicly available genome-wide association studies (GWAS) catalog ^8^. We generated a list of the top ~30 SNPs most strongly associated with vitamin D (with p-value < 5x10^-8^) and their corresponding genes. With this SNP list, we searched for scientific papers reporting the association of these SNPs or genes with vitamin D concentration in the Brazilian population. We consulted the databases of PubMed (9), Literatura Latino-Americana e do Caribe em Ciências da Saúde (LILACS) (10), Scopus ^11^, Scientific Electronic Library Online (SciELO) (12), and Biblioteca Digital Brasileira de Teses e Dissertações (BDTD) ^13^. The search was carried out using the reference SNPs cluster ID (rsID) or the name of the gene where the SNPs is located, together with the terms "Brazil" and "vitamin D". In addition, we included SNPs located in the vitamin D receptor *(VDR)* gene, extensively studied in populations across the world. We selected studies where the association of genotypes with circulating vitamin D was ascertained and written in English, Portuguese, or Spanish.

From the chosen papers, we extracted the following information: SNPs effect on vitamin D levels, the effect allele, allele frequencies, sample size, prevalence of vitamin D deficiency and insufficiency, female percentage, mean age, white ethnicity percentage, study type, Hardy-Weinberg equilibrium test, adjustment for population stratification, and target population.

## Results

Twenty-eight SNPs strongly associated with vitamin D in blood, mainly in European populations, were obtained from the GWAS catalog (table 1). Also, we considered 18 extra SNPs in the *VDR* gene (table 2).

**Table 1:** Single nucleotide polymorphisms associated with vitamin D levels found in the GWAS catalog

Variant	Risk allele	p-value	Risk allele frequency (RAF)	Beta	95%Cl	Mapped gene	Chr	Location (bp) GRCh38	Study accession
rs145432346	C	7x10-286	0.826	0.108 unit increase	[0.10-0.11]	GC	4	71709300	GCST90019543
rs2282679	C	2x10-49	0.260	0.380 unit decrease	[0.32-0.44]	GC	4	71742666	GCST000664
rs2282679		2x10-14	0.290	No data	No data	GC	4	71742666	GCST001560
rs2282679	T	1x10-187	No data	No data	No data	GC	4	71742666	GCST005366
rs2282679	T	4x10-63	0.770	16.628 (z-score) increase	No data	GC	4	71742666	GCST005782
rs2282679	T	5x10-62	No data	No data	No data	GC	4	71742666	GCST005782
rs3755967	T	5x10-343	No data	0.089 unit decrease	[0.084-0.094]	GC	4	71743681	GCST005367
rs3755967		1x10-300	No data	0.206 unit decrease	[0.20-0.21]	GC	4	71743681	GCST90000618
rs3755967	T	5x10-343	No data	0.089 unit decrease	[0.084-0.094]	GC	4	71743681	GCST90019526
rs3755967		1x10-300	No data	0.206 unit decrease	[0.20-0.21]	GC	4	71743681	GCST90019540
rs11723621	G	3x10-1689	0.291	0.186 unit decrease	[0.18-0.19]	GC	4	71749645	GCST90019526
rs1352846	A	1x10-300	0.709	0.194 unit decrease	[0.19-0.20]	GC	4	71752058	GCST90019527
rs1352846	G	1x10-300	0.290	0.233 unit increase	[0.23-0.24]	GC	4	71752058	GCST90019528
rs1352846	G	1x10-300	0.290	0.188 unit increase	[0.18-0.20]	GC	4	71752058	GCST90019532
rs1352846	A	1x10-300	0.709	0.193 unit increase	[0.19-0.20]	GC	4	71752058	GCST90019534
rs1352846	G	1x10-297	0.290	0.121 unit decrease	[0.12-0.13]	GC	4	71752058	GCST90019541
rs4588	T	2x10-263	0.283	0.25 nmol/L decrease	[0.23-0.27]	GC	4	71752606	GCST90019546
rs7041	C	1x10-7	0.170	5.3 (z-score) increase	No data	GC	4	71752617	GCST005782
rs3775150	C	4x10-295	0.262	0.090 unit decrease	[0.086-0.096]	GC	4	71775033	GCST90019542
rs10832254	G	1x10-320	0.370	0.132 unit increase	[0.13-0.14]	RRAS2, COPB1	11	14413152	GCST90019526
rs10832254	G	1x10-300	0.370	No data	No data	RRAS2, COPB1	11	14413152	GCST90019533
rs577185477	C	2x10-342	0.015	0.379 unit decrease	[0.36-0.40]	PSMA1	11	14591017	GCST90019526
rs10832289	T	2x10-266	0.410	0.068 unit decrease	[0.065-0.072]	PDE3B	11	14647950	GCST90019545
rs188480917	G	5x10-275	0.011	0.343 unit decrease	[0.32-0.36]	PDE3B	11	14764324	GCST90019544
rs116970203	No data	1x10-300	No data	0.365 unit decrease	[0.35-0.38]	PDE3B	11	14855172	GCST90019529
rs116970203	G	1x10-300	0.973	0.376 unit increase	[0.36-0.39]	PDE3B	11	14855172	GCST90019535
rs116970203	G	1x10-300	0.973	0.377 unit decrease	[0.37-0.39]	PDE3B	11	14855172	GCST90019537
rs1894100		1x10-300	No data	0.102 unit decrease	[0.097-0.107]	ACTE1P	11	14855172	GCST90019530
rs117913124	A	2x10-775	0.028	0.354 unit decrease	[0.34-0.37]	CYP2R1	11	14879385	GCST90019526
rs12794714	G	1x10-300	0.578	0.0878 unit increase	[0.084-0.092]	CYP2R1	11	14892029	GCST90019536
rs12794714	G	1x10-300	0.578	0.089 unit decrease	[0.085-0.093]	CYP2R1	11	14892029	GCST90019538
rs10741657	A	2x10-38	No data	No data	No data	CALCB, CYP2R1	11	14893332	GCST005366
rs10741657	A	2x10-46	No data	0.031 unit increase	[0.027-0.035]	CALCB, CYP2R1	11	14893332	GCST005367
rs10741657	A	2x10-6	No data	No data	No data	CALCB, CYP2R1	11	14893332	GCST005782
rs10741657	A	3x10-11	0.421	2.1 mmol/L increase	No data	CALCB, CYP2R1	11	14893332	GCST012014
rs11023379		5x10-226	No data	0.065 unit decrease	[0.061-0.069]	CALCB	11	14908414	GCST90019549
rs11233933		1x10-300	No data	0.115 unit decrease	[0.11-0.12]	NADSYN1	11	71419297	GCST90019531
rs12803256	G	9x10-407	0.771	0.100 unit increase	[0.096-0.105]	ACTE1P	11	71421822	GCST90019526
rs12803256	A	1x10-300	0.223	0.105 unit decrease	[0.10-0.11]	ACTE1P	11	71421822	GCST90019539
rs12785878	T	4x10-62	No data	0.0360 unit increase	[0.032-0.040]	NADSYN1	11	71456403	GCST005367
rs12800438	A	1x10-16	No data	No data	No data	NADSYN1	11	71459957	GCST005782
rs4944957	A	1x10-16	No data	No data	No data	NADSYN1	11	71459957	GCST005782
rs12278461	C	5x10-228	0.210	0.129 unit decrease	[0.12-0.14]	NADSYN1	11	71471139	GCST90019548
rs3829251	A	3x10-9	0.190	0.180 unit decrease	[0.12-0.24]	NADSYN1	11	71483513	GCST000664
rs200454003	T	4x10-256	0.265	0.086 unit decrease	[0.082-0.092]	NADSYN1	11	71517944	GCST90019547
rs10745742	T	1x10-7	No data	No data	No data	AMDHD1	12	95964751	GCST005366
rs10745742	T	2x10-20	No data	0.019 unit increase	[0.015-0.023]	AMDHD1	12	95964751	GCST005367
rs17216707	T	1x10-14	No data	No data	No data	CYP24A1, BCAS1	20	54115823	GCST005366
rs17216707	T	8x10-23	No data	0.026 unit increase	[0.021-0.031]	CYP24A1, BCAS1	20	54115823	GCST005367
rs17216707	T	6x10-48	0.817	0.038 unit decrease	[0.032-0.044]	CYP24A1, BCAS1	20	54115823	GCST90000616

bp: base pairs; Chr: chromosome

Same single nucleotide polymorphisms identified in different studies are shown in colour.

**Table 2:** VDR single nucleotide polymorphisms examined in relation to vitamin D levels in the Brazilian population

Variant	Allele 1	Allele 2	Chromosome	Location (bp) GRCh38	Gene position
rs9729	C	A	12	47842840	3’UTR
rs739837	G	T	12	47844438	3’UTR
rs731236	G	A	12	47844974	Ile352Ile
rs7975232	C	A	12	47845054	intron
rs1544410	T	C	12	47846052	intron
rs7963776	G	A	12	47849594	intron
rs7967152	A	C	12	47850401	intron
rs2189480	G	T	12	47870045	intron
rs2228570	A	G	12	47879112	Met1Thr
rs2853564	C	T	12	47884704	intron
rs7965274	T	C	12	47886384	intron
rs2853561	C	T	12	47887474	intron
rs10875694	T	A	12	47887877	intron
rs59128934	G	T	12	47891025	intron
rs11168287	G	A	12	47891631	intron
rs4328262	G	T	12	47891865	intron
rs4237855	G	A	12	47893420	intron
rs11568820	A	G	12	47908762	-

bp: base pairs

### GC vitamin D binding protein gene

Vitamin D binding protein gene (GC) is located on chromosome 4q13.3 and encodes for the VDBP. Nine SNPs in this gene were among the 28 variants most robustly associated with serum vitamin D in previous GWAS (i.e. rs11723621, rs1352846, rs145432346, rs222020, rs2282679, rs3755967, rs3775150, rs4588, rs7041). Only rs2282679, rs4588, and rs7041 were analyzed in the Brazilian population (supplementary table 1). We found a total of six published studies in Brazil, three in Porto Alegre, the capital of the state of Rio Grande do Sul, and one each in the states of Rio de Janeiro, Paraná, and São Paulo. The target populations were diverse and involved women of reproductive age, university civil servants, and individuals affected by chronic diseases such as hepatitis C and cirrhosis, but their minor allele frequencies were quite similar (table 3).

**Table 3:** Allele frequencies of GC single nucleotide polymorphisms tested in association with vitamin D levels in the Brazilian population

rs4588	City, State	Allele 1	Allele 2	Allele 1 frequency
Adult patients with chronic hepatitis C genotype 1	Porto Alegre, RS	T	G	0.213
Women with no evidence of clinical disease	Porto Alegre, RS	A	C	0.293
Women of reproductive age	Porto Alegre, RS	A	C	0.230
Healthy female students	Curitiba, PR	A	C	0.267
Patients with cirrhosis	São Jose do Rio Preto, SP	A	C	0.300
Controls (cirrhosis)	São Jose do Rio Preto, SP	A	C	0.280
rs7041				
Adult patients with chronic hepatitis C genotype 1	Porto Alegre, RS	C	A	0.461
Women with no evidence of clinical disease	Porto Alegre, RS	G	T	0.484
Women of reproductive age	Porto Alegre, RS	G	T	0.535
Healthy female students	Curitiba, PR	G	T	0.485
Patients with cirrhosis	São Jose do Rio Preto, SP	G	T	0.460
Controls (cirrhosis)	São Jose do Rio Preto, SP	G	T	0.510
rs2282679				
University civil servants	Rio de Janeiro, RJ	C	A	0.222
Women with no evidence of clinical disease	Porto Alegre, RS	C	A	0.283
Women of reproductive age	Porto Alegre, RS	C	A	0.225

PR: Paraná; RJ: Rio de Janeiro: RS: Rio Grande do Sul; SP: São Paulo

Overall, we uncovered evidence of the *GC* gene associated with vitamin D concentrations in Brazil, with the rs4588 A allele, the rs7041 T allele, and the rs2282679 C allele underlying lower vitamin D levels.

### Vitamin D receptor gene (VDR)

Despite not being one of the genes identified in earlier GWAS as associated with vitamin D levels, the Vitamin D receptor gene *(VDR)* has been examined in numerous human groups, often in studies conducted before the GWAS era. Our literature search identified 12 publications assessing circulating vitamin D with *VDR* genotypes in Brazil (supplementary table 2). The SNPs rs1544410 (G/A), rs2228570 (C/T), rs731236 (T/C), and rs7975232 (T/G), formerly detected using the restriction enzymes BsmI, FokI, TaqI, and ApaI, respectively, were ascertained in most analyses, encompassing a variety of populations across the country (table 4). However, unlike what was observed with the GC gene, results were inconsistent in terms of the effect found or the direction of that effect. For instance, while the A allele of SNPs rs1544410 was associated with lower levels of vitamin D in young children from Acre ^14^, it increases vitamin D in girls 7-18 years old from south Brazil ^15^. The C allele at SNPs rs731236 was associated with higher serum vitamin D in girls from south Brazil and pregnant women from Bahia but appeared to have the opposite effect in type 1 diabetes patients from Pará state ^16^.

**Table 4:** Allele frequencies of VDR single nucleotide polymorphisms most frequently tested in association with vitamin D levels in the Brazilian population

rs1544410 (BsmI)	City, State	Allele 1	Allele 2	Allele 1 Frequency
Adolescents without chronic disease	João Pessoa, PB	A	G	0.395
Adult male patients with Chagas disease	Botucatu, SP	A	G	0.400
Patients with polycystic ovary syndrome	Porto Alegre, RS	A	G	0.400
Non-hirsute women with regular ovulatory cycles	Porto Alegre, RS	A	G	0.350
Children aged ≤ 10 years	Acrelândia, AC	T	C	0.406
Healthy girls	Curitiba, PR/Porto Alegre, RS	A	G	0.323
Type 1 diabetes (T1D) patients	Belém, PA	A	G	n/a
Controls (T1D)	Belém, PA	A	G	n/a
Type 2 diabetes (T2D) patients	Belo Horizonte, MG	A	G	0.401
Controls (T2D)	Belo Horizonte, MG	A	G	0.411
Colorectal cancer cases	São Paulo, SP			n/a
Controls (CRC)	São Paulo, SP			n/a
rs2228570 (FokI)				
Children with persistent primary teeth (PPT)	Riberão Preto, SP	A	G	0.250
Controls (PPT)	Riberão Preto, SP	A	G	0.269
Children with delayed tooth eruption (DTE)	Riberão Preto, SP	A	G	0.296
Controls (DTE)	Riberão Preto, SP	A	G	0.379
Adolescents without chronic disease	João Pessoa, PB	T	C	0.332
Adult male patients with Chagas disease	Botucatu, SP	T	C	0.440
Children aged ≤ 10 years	Acrelândia, AC	A	G	0.299
T1D patients	Belém, PA	T	C	0.308
Controls (T1D)	Belém, PA	T	C	0.331
T2D patients	Belo Horizonte, MG	T	C	0.245
Controls (T2D)	Belo Horizonte, MG	T	C	0.306
rs731236 (TaqI)				
Pregnant women	Santo Antônio de Jesus, BA	G	A	0.300
Adult male patients with Chagas disease	Botucatu, SP	G	A	0.260
Patients with PCOS	Porto Alegre, RS	G	A	0.396
Non-hirsute women with regular ovulatory cycles	Porto Alegre, RS	G	A	0.354
Children aged ≤ 10 years	Acrelândia, AC	G	A	0.396
Healthy girls	Curitiba, PR/Porto Alegre, RS	C	T	0.314
T1D patients	Belém, PA	C	T	0.315
Controls (T1D)	Belém, PA	C	T	0.283
T2D patients	Belo Horizonte, MG	C	T	0.332
Controls (T2D)	Belo Horizonte, MG	C	T	0.403
rs7975232 (ApaI)				
Pregnant women	Santo Antônio de Jesus, BA	C	A	0.400
Patients with PCOS	Porto Alegre, RS	C	A	0.447
Non-hirsute women with regular ovulatory cycles	Porto Alegre, RS	C	A	0.400
Children aged ≤ 10 years	Acrelândia, AC	C	A	0.431
Healthy girls	Curitiba, PR/Porto Alegre, RS	G	T	0.429
T1D patients	Belém, PA	G	T	0.362
Controls (T1D)	Belém, PA	G	T	0.446
T2D patients	Belo Horizonte, MG	C	A	0.245
Controls (T2D)	Belo Horizonte, MG	C	A	0.210
Colorectal cancer (CRC) cases	São Paulo, SP			n/a
Controls (CRC)	São Paulo, SP			n/a
rs739837				
Children with persistent primary teeth (PPT)	Riberão Preto, SP	G	T	0.400
Controls (PPT)	Riberão Preto, SP	G	T	0.500
Children with delayed tooth eruption (DTE)	Riberão Preto, SP	G	T	0.417
Controls (DTE)	Riberão Preto, SP	G	T	0.366
SCAALA cohort	Salvador, BA	G	T	0.484
rs11568820 (Cdx2)				
Children aged ≤ 10 years	Acrelândia, AC	A	G	0.399
Asthmatic children	Curitiba, PR	A	G	0.284
Non-asthmatic children	Curitiba, PR	A	G	0.295

AC: Acre; BA: Bahia; MG: Minas Gerais; PA: Pará; PB: Paraíba; PR: Paraná; RS: Rio Grande do Sul; SP: São Paulo

n/a: not applicable

### Other genes

We identified 25 SNPs in eight genes other than *GC* and *VDR* among the top predictors of vitamin D levels in the GWAS catalog. However, just four of these genes have been explored in Brazil *(CYP2R1, CYP24A1, CYP27B1, NADSYN1* ) (supplementary table 3). Several polymorphisms in *CYP2R1* and *CYP24A1* were associated with serum vitamin D and vitamin D insufficiency in a study of ~800 young people from deprived areas in Salvador, Bahia ^17^. In contrast, smaller studies investigating the same genes, but different SNPs and populations did not find any effect ^18^^-^^20^.

## Discussion

Despite the widespread availability of sunlight across Brazil and UVR levels ensuring vitamin D synthesis in the skin ^21^, numerous Brazilian studies report a high prevalence of vitamin D deficiency and insufficiency ^22^. Since 2017, the *Sociedade Brasileira de Endocrinologia e Metabologia* (SBEM) and the *Sociedade Brasileira de Patologia Clínica/ Medicina Laboratorial*(SBPC/ML) recommend a 25OHD level equal to or above 20 ng/ml for individuals up to 60 years old, and a range of 30 to 60 ng/ ml for at-risk groups ^23^. Considering other sources of vitamin D like diet and supplementation, vitamin D intake in Brazil is limited, food fortification is uncommon, and the use of vitamin D supplements (≤ 10%) is infrequent ^23^. To that extent, the SBEM only recommends supplementation for specific groups at risk of deficiency, for example, pregnant and lactating women, individuals with osteoporosis, elderly people, and patients with conditions that affect vitamin D metabolism ^24^.

In general, our findings showed limited local research on the genetic determinants of vitamin D levels, with a predilection towards investigating the *VDR* gene, but with sounder evidence accumulating on the effects of the *GC* gene. This observation agrees with the GWAS data indicating that *GC,* the gene that encodes for the binding protein, is among the dominant genetic predictors of vitamin D concentrations in European, Asian, and African-ancestry populations ^25^^-^^30^. Conversely, a look-up of *VDR* in the GWAS catalog returned associations with different traits but not with vitamin D levels (supplementary table 4).

Brazil needs to conduct more research to confirm the role of *GC* (and clarify the one of *VDR)* and to reveal other genetic variants robustly associated with serum vitamin D. The identification of reliable proxies will allow us to establish causal associations with disease and promote the use of appropriate polygenic risk scores for predictive purposes.

Additionally, we would like to suggest a few improvements to future studies, especially to use them as the basis for meta-analyses. For example, it is important to describe all findings (significant and non-significant) and to provide them as supplementary material, if necessary, assess Hardy-Weinberg equilibrium and report test results, and, given Brazil's admixed genetic background, adjust for markers of population stratification or related variables *(e.g.,* race/ethnicity, socioeconomic status) when these are unavailable.

Among the limitations of our study, there is still the chance that we have missed relevant publications not covered by our search parameters, or SNPs associated with vitamin D in the GWAS catalog, outside the top 30, with reports in Brazilian populations, although this is rather unlikely. In addition, given the limited number of studies found and the heterogeneity of the included samples, it was not possible to run a meta-analysis to obtain an indication of the strength and direction of the effect of *GC* variants on the levels of vitamin D, making unfeasible the implementation of any action in clinical practice linked to our results.

In conclusion, there is a lot of interest in vitamin D as a potential risk factor for several chronic diseases of public health impact. Therefore, it is essential to identify causal relationships between vitamin D levels and disease outcomes. One way of improving causal inference would be to apply Mendelian randomization, which uses genetic variants to proxy or instrument the exposure *(e.g.,* serum vitamin D) to obtain unbiased estimates of these relationships. However, the instruments should be appropriate for the study population, either having been discovered or validated locally. We noticed insufficient research in Brazil (and South America) on vitamin D proxies, with existing studies focusing on the *VDR* as a genetic risk factor for disease, which may or may not produce changes in circulating vitamin D.

## Supplementary archives

**Supplementary table 1:** Scientific articles on *GC* polymorphisms and vitamin D levels in the Brazilian population

study	database	authors	acess link	population	region/city/town	gene	SNP	effect or minor allele	effect or minor allele frequency	prevalence of vitamin D deficiency (95% CI)	prevalence of vitamin D insuficiency (%)	p-value	25OHD (ng/ml) mean ± sd	p-value	beta 25OHD (ng/ml)	CI 95%/se	p-value	OR	CI 95%	p-value	DBP (ug/ml)	p-value	N	age (years) mean ± sd (range)	% female	ethnicity (% whites)	Hardy-Weinberg equilibrium (p-value)	correction for population stratification	type of study	comments
Genetic, sociodemographic and lifestyle factors associated with serum 25-hydroxyvitamin D concentrations in Brazilian adults: the Pró-Saúde Study	PUBMED	Bezerra et al. (2022)	https://pubmed.ncbi.nlm.nih.gov/35043885/	university civil servants	Rio de Janeiro, RJ	GC	rs2282679	C	0,222		55,0	<0.001	48.0 ± 19.1 nmol/l	<0.001									491	45-54 (43.8%)	51,1		in equilibrium	no	cross-sectional	
								CC			65,4		38.6 (27.2)		-10,63	(-17.52, -3.74)														
								CA			65,7		44.4 (21.9)		-6,84	(-10.09, -3.59)														
								AA			48,2		50.3 (28.3)		reference															
Genetic polymorphisms related to the vitamin D pathway in patients with cirrhosis with or without hepatocellular carcinoma (HCC)	PUBMED	Brait et al. (2022)	https://pubmed.ncbi.nlm.nih.gov/35919232/	patients with cirrhosis & controls	São Jose do Rio Preto, SP	GC	rs4588; rs7041	A; G	rs4588 A: 0.30 cases/0.28 controls		30.0 cases/35.0 controls												383	16-81; 20-84	21.5; 43.6		in equilibrium in both cirrhosis cases and controls	no	case-control	reduced levels of vitamin D in cases showed association with genotypes with at least one mutant allele (_/A) for GC-rs4588 (77.8%) compared to controls (14.3%; p = 0.0406).
									rs7041 G: 0.46 cases/0.51 controls																					
Effect of vitamin D serum levels and GC gene polymorphisms in liver fibrosis due to chronic hepatitis C	PUBMED	Azevedo et al. (2017)	https://pubmed.ncbi.nlm.nih.gov/28809744/	adult patients with chronic hepatitis C genotype 1	Porto Alegre, RS	GC	rs4588; rs7041	T; C	0.213; 0.461	50,0	27,3	0,02	19.9 (14.0-29.4)										132	53 (± 9)	46,2		in equilibrium	no	cross-sectional	25OHD levels differences between haplotypes too.
							rs4588	GG + GT					20.2 (15.8-29.8)	0,023																
								TT					9.6 (8.1-20.9)																	
							rs7041	CC + CA					22.8 (16.2-30.1)	0,026																
								AA					17.0 (8.9-26.6)																	
Prevalence of vitamin D deficiency in women from southern Brazil and association with vitamin D-binding protein levels and GC-DBP gene polymorphisms	PUBMED	Santos et al. (2019)	https://pubmed.ncbi.nlm.nih.gov/31830090/	women with no evidence of clinical disease	Porto Alegre, RS	GC	rs4588; rs7041; rs2282679	A; G; C	0.293; 0.484; 0.283	39,7			22.80 (± 8.32)										443	53.4 (± 9.4)	100	80	0.23, 0.09, 0.68	no	cross-sectional	
							rs4588	CC					23.00 ± 8.84	0,282	-0,6						202.98 ± 28.28	< 0.001								
								CA					23.16 ± 7.83								196.49 ± 29.88									
								AA					20.77 ± 7.16								183.95 ± 36.85									
							rs7041	TT					21.48 ± 7.54	0,030	1.2						192.96 ± 33.41	0,078								
								TG					23.20 ± 8.25								201.38 ± 28.06									
								GG					23.78 ± 9.14								199.82 ± 30.02									
							rs2282679	AA					23.39 ± 8.79	0,034	-1,3			1,00			203.13 ± 27.90	< 0.001								
								AC					22.83 ± 7.81					0,981	(0.758; 1.269)	0,884	196.41 ± 30.04									
								CC					19.70 ± 7.17					1,740	(1.301; 2.237)	<0.001	180.88 ± 38.20									
Genetic variant in vitamin D-binding protein is associated with metabolic syndrome and lower 25-hydroxyvitamin D levels in polycystic ovary syndrome: A cross-sectional study	PUBMED	Santos et al. (2017)	https://pubmed.ncbi.nlm.nih.gov/28278285/	women of reproductive age	Porto Alegre, RS	GC	rs4588; rs7041; rs2282679	A; G; C	0.230; 0.535; 0.225	42,2	45,1		21.48 ± 7.25; 21.50 ± 6.90										291 (191 PCOS + 100 controls)/102 (54 PCOS + 48 controls) with 25OHD levels	22.89 ± 6.66 PCOS/25.18 ± 7.72 controls	100	93.9	in equilibrium in both PCOS and control groups	no	cross-sectional	LD rs4588 & rs7041 r2 = 0.44
							rs4588	CC				0,542																		
								CA + AA																						
							rs7041	TT		69,6		0,002																		
								TG + GG		exact value not available																				
							rs2282679	AA				0,542																		
								AC + CC																						
Variations in the vitamin D-binding protein (DBP) gene are related to lower 25-hydroxyvitamin d levels in healthy girls: a cross-sectional study	PUBMED	Santos et al. (2013)	https://pubmed.ncbi.nlm.nih.gov/23548751/	healthy female students	Curitiba, PR	GC	rs4588; rs7041	A; G	0.267; 0.485				22.1 ± 5.9										198	13.17 ± 1.74	100		in equilibrium	no	cross-sectional	LD rs4588 & rs7041 r2 = 0.38
							rs4588	CC						0,030	-1,65		0,012	2,73	(0.94; 7.93)			A allele								25OHD levels differences between haplotypes too.
								CA																						
								AA																						
							rs7041	TT						0,010	-1,74		0,002	3,47	(1.45; 8.27)			T allele								
								TG																						
								GG																						

**Supplementary table 2:** Scientific articles on *VDR* gene polymorphisms and vitamin D levels in the Brazilian population

Study	Database	Authors	Access link	Population	Region/city/town	Gene	SNP	Effect or minor allele	Effect or minor allele frequency	Prevalence of vitamin D deficiency (95% CI)	p-value	Prevalence of vitamin D insuficiency (%)	p-value	25OHD (ng/ml) mean ± sd	p-value	N	Age (years) mean ± SD (range)	% female	Ethnicity (% white)	Beta 25OHD (ng/mL)	95% CI/SE	p-value	OR	CI 95%	p-value	Hardy-Weinberg equilibrium (p-value)	Correction for population stratification	Type of study	Comments
Vitamin D deficiency is a risk factor for delayed tooth eruption associated with persistent primary tooth (PPT)	PUBMED	Xavier et al. (2021)	https://pubmed.ncbi.nlm.nih.gov/33944665/	children with primary teeth with exfoliation time expired for more than a year (persistent primary tooth) and children with regular primary teeth exfoliation time (controls)	Riberao Preto, SP	VDR	rs2228570; rs739837	A; G		26.7 PPT/0.0 controls				14.2-37.4 PPT/21.9-48.2 controls		30 (15 PPT + 15 controls)	9.4 ± 1.8	43,3								not reported	no	case-control	data showed no association between genetic polymorphisms in VDR and serum 25OHD levels (p>0.05).
							rs2228570	A	0.269 controls/0.350 PPT; 0.379 controls/0.296 DTE																				
							rs739837	G	0.500 controls/0.400 PPT; 0.366 controls/0.417 DTE																				
Genetic polymorphisms in vitamin D pathway infuence 25(OH)D levels and are associated with atopy and asthma	PUBMED	Galvão et al (2020)	https://pubmed.ncbi.nlm.nih.gov/32834827/	SCAALA cohort (children from deprived areas)	Salvador, BA	VDR	rs10875694; rs11168287; rs2189480; rs2853561; rs2853564; rs4237855; rs4328262; rs59128934; rs739837; rs7963776; rs7965274; rs7967152; rs9729	G; C; G; G; G; G; T; A; C		20,8		40,7		27.33 ± 9.60		792	(11-19)	47,6								in equilibrium	individual genetic ancestry using 269 AIMs	cross-sectional nested in cohort	write to them to ask the DBP (GC) SNPs vs 25OHD levels.
							rs11168287	G	0,377														0,78	0.63, 0.97	0,028				
							rs2853564	C	0,177														1,30	1.00, 1.70	0,049				
							rs4237855	G	0,323														0,79	0.63, 0.99	0,038				
							rs59128934	G	0,041														2,07	1.28, 3.34	0,002				
							rs739837	G	0,484														0,78	0.63, 0.96	0,019				
							rs7963776	G	0,474														0,79	0.64, 0.98	0,029				
							rs7965274	T	0,180														1,31	1.01, 1.70	0,044				
							rs7967152	A	0,461														0,77	0.62, 0.95	0,013				
							rs9729	C	0,480														0,78	0.70, 0.96	0,017				rs9729 C allele increases VDR expression (p = 0.0007) in GTEx. Rs9729 is in strong LD with rs731236 (TaqI).
							rs59128934	G	0,057														1,78	1.12, 2.83	0,014				
Variants rs1544410 and rs2228570 of the vitamin D receptor gene and glycemic levels in adolescents from Northeast Brazil	PUBMED	Neves et al. (2019)	https://pubmed.ncbi.nlm.nih.gov/31718198/	adolescents who did not present any chronic disease	João Pessoa, PB	VDR	rs1544410; rs2228570	B = A; f = T	0.395; 0.332			50,0		28.0 (28.4-30.7)		208	17.7 (± 1.14)	62,5								rs1544410 out of HWE (calculated by us)	no	cross-sectional	
							rs1544410	BB = AA						31.23 (9.35)	0,281	47							1,72	0.84, 3.50	0,134				
								Bb = GA						29.12 (7.80)		70							1,01	0.54, 1.90	0,967				
								bb = GG						29.01 (7.90)		91							1,00						
							rs2228570	FF = CC						29.23 (7.76)	0,840	92													
								Ff = TC						30.03 (9.06)		94													
								ff = TT						28.81 (6.90)		22													
Polymorphism in the vitamin D receptor gene is associated with maternal vitamin D concentration and neonatal outcomes: A Brazilian cohort study	PUBMED	Pereira Santos et al. (2019)	https://pubmed.ncbi.nlm.nih.gov/31070844/	pregnant women who lived in the urban area of the municipality and received prenatal services	Santo Antônio de Jesus, BA	VDR	rs731236; rs7975232	G; C	0.300; 0.400	23,0		43,0		72.62 ± 31.51 nmol/l		270	26.73 ± 5.85	100	18,15	nmol/L						0.24; 0.94	no	prospective cohort	
							rs731236	GG vs AA												14,09	0.85, 27.34	0,03							
							rs7975232	CC vs AA												1,15	(-10.28, 12.59)	0,84							
Association of vitamin D3, VDR gene polymorphisms. and LL-37 with a clinical form of Chagas Disease	SciELO	Junior et al. (2019)	https://www.scielo.br/j/rsbmt/a/z7QwDmFg7Ndz6RxRtbJG8SK/?lang=en	adult male patients with indeterminate and cardiac form of chronic Chagas Disease (CD)	Botucatu, SP	VDR	rs1544410; rs2228570; rs731236; rs7975232		0.40; 0.44; 0.26; 0.31	10,9		53,1	0.207; 0.767; 0.617; 0.837	29.3 ± 5.8; 25.4 ± 7.3		64 (46 indeterminate + 18 cardiac)	60.3 ± 8.1; 62.2 ± 11.0	0	76,6							not reported	no	cross-sectional	
Apa-I polymorphism in VDR gene is related to metabolic syndrome in polycystic ovary syndrome: a cross-sectional study	PUBMED	Santos et al. (2018)	https://pubmed.ncbi.nlm.nih.gov/29669566/	patients with polycystic ovary syndrome (PCOS) + non-hirsute women with regular ovulatory cycles	Porto Alegre, RS	VDR	rs1544410; rs731236; rs7975232	A; G; C	0.400/0.350; 0.396/0.354; 0.447/0.400					21.47 ± 7.61; 21.50 ± 6.90		291 (191 PCOS + 100 controls)	22.89 ± 6.66; 25.18 ± 7.72									in equilibrium		cross-sectional	
							rs7975232	AA + CA						21.52 ± 7.16	0,399														
								CC						21.31 ± 6.15									as						
Mutações do gene receptor da vitamina D e níveis séricos de vitamina D em crianças com asma	SciELO	Santos et al. (2018)	https://www.scielo.br/j/rpp/a/wVLMgrfnLrBbDVsCdLFcvCJ/?lang=pt	children aged 7 to 14 years (asthmatics and non-asthmatics)	Curitiba, PR	VDR	Cdx2 (rs11568820?)	G	0.714 (0.716 asthmatics/0.705 non-asthmatics)			98,0				77 (60 asthmatic + 17 non-asthmatic)	10.8 ± 2.2	43,0								not reported	no	cross-sectional	There was no association between vitamin D, PTH or calcium levels with any of the polymorphisms studied.
Genetic polymorphisms of vitamin D metabolism genes and serum level of vitamin D in colorectal cancer	PUBMED	Vidigal et al. (2017)	https://pubmed.ncbi.nlm.nih.gov/28665452/	colorectal cancer cases & controls	São Paulo, SP	VDR	rs1544410; rs7975232			51.0; 43.3		18.4; 26.6		26.4 ± 17.6; 28.4 ± 19.2		473 (152 CRC + 321 controls)	62.8 ± 13.0; 62.7 ± 10.4	46.7; 49.2								not reported	no	case-control	
							rs1544410	AA						29.5; 24.4	0,482														
								Aa + aa						27.1; 28.3	0,074														
							rs7975232	BB						32.5; 23.2	0,216														
								Bb + bb						27.1; 27.5	0,155														
25-hydroxyvitamin D3 levels, BsmI polymorphism and insulin resistance in Brazilian Amazonian children	PUBMED	Cobayashi et al. (2015)	https://pubmed.ncbi.nlm.nih.gov/26047339/	children aged ≤ 10 years	Acrelândia, AC	VDR	rs11568820; rs1544410; rs2228570; rs731236; rs7975232	A; T; A; G; C	0.399; 0.406; 0.299; 0.396; 0.431	11.1 (9.2-13.2)		21.8 (19.2-24.5)		66 (86-105)		1225 (974 with 25OHD levels)	5.4 ± 2.8 (2.8 months-10.4 years)	49,0	10,3	0.007; 0.100						HWE was tested, results not reported.	adjusted for race/ethnicity		
							rs1544410	T												-0,070	-0.132. -0.008	0,026							
							rs1544410	T												-0,053	-0.100. -0.006	0,025	adjusted for sex, age, race/ethn						
Vitamin D deficiency in girls from South Brazil: a cross-sectional study on prevalence and association with vitamin D receptor gene variants	PUBMED	Santos et al (2012)	https://pubmed.ncbi.nlm.nih.gov/22681928/	healthy girls	Curitiba, PR/Porto Alegre, RS	VDR	rs1544410; rs731236; rs7975232	A; C; G		36,3		54,3		21.3 ± 6.8		234	13.0 ± 1.9 (7-18)	100,0								in equilibrium	no	cross-sectional	LD: rs1544410 & rs7975232 r2=0.330/rs1544410 & rs731236 r2=0.807/rs7975232 & rs731236 r2=0.319
							rs1544410	A	0,323	GA + AA vs GG	0,014									3,114	0,881	< 0.001	1,96	1.14, 3.37					there were differences in serum 25OHD levels by haplotype as well
							rs731236	C	0,314	TC + CC vs TT	0,034									2,505	0,890	0,005	1,78	1.04, 3.06					
							rs7975232	G	0,429	GT + TT vs GG	0,078									2,575	1,189	0,031		0.27, 1.08					
Variants in the VDR gene may influence 25(OH)D levels in type 1 diabetes mellitus in a Brazilian population		Ferraz et al. (2022)	https://pubmed.ncbi.nlm.nih.gov/35267984/	T1D patients & controls	Belém, PA	VDR	rs1544410; rs2228570; rs731236; rs7975232	A; T; C; T	na/na; 0.308/0.331; 0.315/0.283; 0.638/0.554					26.04 ± 8.45; 32.60 ± 8.85		148 (65 T1D + 83 controls)	27.3 ± 10.4; 38.5 ± 13.6	53.9; 77.1								in equilibrium in both cases and controls	61 AIMs used to estimate individual ancestry but no correction made	case-control	
							rs1544410	AA						lower levels than GG + GA	< 0.05														
							rs2228570	TT						higher levels than CC + CT	< 0.05														
							rs731236	CC						lower levels than TT + TC	< 0.05														
							rs7975232								n.s														
Lower vitamin D levels, but not VDR polymorphisms, influence type 2 diabetes mellitus in Brazilian population independently of obesity		Rodrigues et al. (2019)	https://pubmed.ncbi.nlm.nih.gov/31121922/	T2D patients & controls	Belo Horizonte, MG	VDR	rs1544410; rs2228570; rs731236; rs7975232	A; T; C; C	0.401/0.411; 0.245/0.306; 0.332/0.403; 0.245/0.210	59.7; 12.0				17.2 ± 16.6; 30.8 ± 16.2		163 (101 T2D + 62 controls)	56 ± 13; 53 ± 18	81.0 (81.2; 80.6)								all > 0.025	no	case-control	
							rs1544410	AA						26.0 (37.6)	0,415														
								AG						24.8 (18.5)															
								GG						21.2 (19.3)															
							rs2228570	TT						23.2 (20.2)	0,764														
								TC						27.4 (24.5)															
								CC						22.9 (35.2)															
							rs731236	CC						20.3 (30.3)	0,222														
								CT						25.6 (23.3)															
								TT						21.4 (19.3)															
							rs7975232	AA						24.7 (22.7)	0,656														
								AC						25.2 (17.5)															
								CC						17.9 (29.9)															

**Supplementary table 3:** Scientific articles on polymorphisms in genes other than *GC* and *VDR* and vitamin D levels in the Brazilian population

study	database	authors	acess link	population	region/city/town	gene	SNP	effect or minor allele	effect or minor allele frequency	prevalence of vitamin D deficiency (95% CI)	prevalence of vitamin D insuficiency (%)	p-value	25OHD (ng/ml) mean ± sd	p-value	beta 25OHD (ng/ml)	CI 95%/se	p-value	OR	CI 95%	p-value	N	age (years) mean ± sd (range)	% female	ethnicity (% whites)	Hardy-Weinberg equilibrium (p-value)	correction for population stratification	type of study	comments
Genetic, sociodemographic and lifestyle factors associated with serum 25-hydroxyvitamin D concentrations in Brazilian adults: the Pró-Saúde Study	PUBMED	Bezerra et al. (2022)	https://pubmed.ncbi.nlm.nih.gov/35043885/	university civil servants	Rio de Janeiro, RJ	CYP2R1; NADSYN1; CYP24A1	rs10741657; rs12785878; rs6013897	G; T; T			55,0		48.0 ± 19.1 nmol/l								491	45-54 (43.8%)	51,1		rs12785878 & rs6013897 in HWD and not analysed	no	cross-sectional	
							rs10741657	G	0,705			0,119	median (IQR)	0,118														
								GG			59,2		46.0 (25.6)															
								GA			49,5		50.0 (28.3)															
								AA			56,8		49.5 (22.8)															
Genetic polymorphisms in vitamin D pathway infuence 25(OH)D levels and are associated with atopy and asthma	PUBMED	Galvão et al (2020)	https://pubmed.ncbi.nlm.nih.gov/32834827/	SCAALA cohort (children from deprived areas)	Salvador, BA	CYP2R1; CYP24A1	rs10500804, rs12794714; rs2245153, rs34043203, rs3886163, rs4809960, rs56229249	G, A; C, A, T, C, G					27.33 ± 9.60								792	(11-19)	47,6		in equilibrium	individual genetic ancestry using 269 AIMs	cross-sectional nested in cohort	write to them to ask the DBP (GC) SNPs vs 25OHD levels.
						CYP2R1	rs10500804	G							-1,37	(-2.40, 0.35)	0,009	1,40	1.11, 1.77	0,006								
								GG					22.25 ± 9.31	0,043														
								GT					25.67 ± 9.25															
								TT					26.68 ± 9.79															
						CYP2R1	rs12794714	A							-1,38	(-2.40, 0.35)	0,009	1,41	1.11, 1.79	0,005								
								AA					24.56 ± 9.28	0,058														
								AG					26.67 ± 9.26															
								GG					27.66 ± 9.73															
						CYP24A1	rs2245153	C										0,79	0.63, 0.99	0,042								
						CYP24A1	rs34043203	A										1,49	1.00, 2.22	0,049								
						CYP24A1	rs3886163	T							-1,48	(-2.77, 0.18)	0,026	1,44	1.05, 1.99	0,025								
								TT					24.49 ± 8.20	0,094														
								TC					25.72 ± 10.00															
								CC					27.41 ± 9.42															
						CYP24A1	rs4809960	C										0,69	0.53, 0.91	0,008								
						CYP24A1	rs56229249	G										1,42	1.04, 1.94	0,028								
Genetic polymorphisms related to the vitamin D pathway in patients with cirrhosis with or without hepatocellular carcinoma (HCC)	PUBMED	Brait et al. (2022)	https://pubmed.ncbi.nlm.nih.gov/35919232/	patients with cirrhosis & controls	São Jose do Rio Preto, SP	CYP24A1	rs6013897	T	0.72; 0.70		30.0; 35.0										383	16-81; 20-84	21.5; 43.6		in equilibrium	no	case-control	no association with vitamin D levels
																												
Genetic polymorphisms of vitamin D metabolism genes and serum level of vitamin D in colorectal cancer	PUBMED	Vidigal et al. (2017)	https://pubmed.ncbi.nlm.nih.gov/28665452/	colorectal cancer cases & controls	São Paulo, SP	CYP24A1; CYP27B1	rs158552; rs17217119; rs6013897; rs10877012	T; A; T; G		51.0; 43.3	18.4; 26.6		26.4 ± 17.6; 28.4 ± 19.2								473 (152 CRC + 321 controls)	62.8 ± 13.0; 62.7 ± 10.4	46.7; 49.2		not reported	no	case-control	
							rs158552	TT					25.7; 28.4	0,199														
								TC + CC					29.7; 25.3	0,385														
							rs17217119	AA					25.8; 25.6	0,247														
								AG + GG					29.9; 24.7	0,660														
							rs6013897	TT					26.9; 26.2	0,493														
								TA + AA					29.0; 26.3	0,958														
							rs10877012	GG					30.2; 26.2	0,047														
								GT + TT					24.0; 28.2	0,381														

**Supplementary table 4:** . Single nucleotide polymorphisms in the *VDR* gene associated with complex traits according to the GWAS catalog

Beta	CI	Mapped gene	Reported trait	Trait(s)	Study accession	Location
0.063426755 unit decrease	[0.042-0.085]	VDR	basal cell carcinoma	basal cell carcinoma	GCST90013410	12:47844438
0.0481096 unit increase	[0.033-0.064]	VDR	total testosterone levels	testosterone measurement	GCST90012112	12:47860570
0.13136138 unit increase	[0.088-0.175]	VDR	medication use (diuretics)	Diuretic use measurement	GCST007928	12:47860570
0.3943 unit increase	[0.28-0.51]	VDR	diastolic blood pressure	diastolic blood pressure	GCST90132904	12:47860570
0.1314 unit increase	[0.088-0.175]	VDR	medication use (diuretics)	diuretic use measurement	GCST90018985	12:47860570
		VDR	cardiovascular disease	cardiovascular disease	GCST007072	12:47860570
		VDR	gout	gout	GCST001356	12:47862166
0.32741 unit increase	[0.18-0.47]	VDR	COVID-19 (hospitalized vs not hospitalized)	COVID-19	GCST90104752	12:47873551
0.75 percent increase		VDR	gut microbiota (beta diversity)	gut microbiome measurement	GCST003876	12:47876015
		VDR	eosinophil counts	eosinophil count	GCST007065	12:47879112
0.0138518615 unit increase	[0.0094-0.0183]	VDR	eosinophil percentage of white cells	eosinophil percentage of leukocytes	GCST90002382	12:47879112
47.572 unit increase		VDR	serum immune biomarker levels	inflammatory biomarker measurement, YKL40 measurement	GCST010146	12:47914289
0.9905315 unit decrease		VDR	sphingomyelin (d32:2) levels	sphingomyelin measurement	GCST90094889	12:47919236
0.57862 unit increase	[0.33-0.83]	VDR	S-6-hydroxywarfarin levels	S-6-hydroxywarfarin measurement	GCST90129565	12:47920142
0.86594 unit increase	[0.52-1.21]	VDR	R-6-hydroxywarfarin to R-warfarin ratio	R-6-hydroxywarfarin to R-warfarin ratio measurement	GCST90129572	12:47927031
		VDR	adolescent idiopathic scoliosis	adolescent idiopathic scoliosis	GCST006287	12:47927916
		VDR, TMEM106C	heel bone mineral density	heel bone mineral density	GCST007066	12:47943286
0.0304 unit increase	[0.024-0.037]	VDR, TMEM106C	glycated hemoglobin levels	HbA1c measurement	GCST90019509	12:47943286
0.0541 unit decrease	[0.038-0.07]	TMEM106C, VDR	glycated hemoglobin levels	HbA1c measurement	GCST90019509	12:47943734
0.049543403 unit increase	[0.032-0.067]	VDR, TMEM106C	medication use (calcium channel blockers)	calcium channel blocker use measurement	GCST007929	12:47944639
		TMEM106C, VDR	red blood cell count	erythrocyte count	GCST007069	12:47952685
0.015703138 unit increase	[0.011-0.02]	VDR, TMEM106C	lymphocyte percentage of white cells	lymphocyte percentage of leukocytes	GCST90002389	12:47963231
